# S100A8/A9 mRNA Induction in an *Ex Vivo* Model of Endotoxin Tolerance: Roles of IL-10 and IFNγ

**DOI:** 10.1371/journal.pone.0100909

**Published:** 2014-06-23

**Authors:** Mathieu Fontaine, Séverine Planel, Estelle Peronnet, Fanny Turrel-Davin, Vincent Piriou, Alexandre Pachot, Guillaume Monneret, Alain Lepape, Fabienne Venet

**Affiliations:** 1 Hospices Civils de Lyon – Intensive Care Unit, Centre Hospitalier Lyon-Sud, Pierre-Bénite, France; 2 Joint Unit Hospices Civils de Lyon – bioMérieux, Hôpital Edouard Herriot, Lyon, France; 3 EAM 4174 “Hemostasis, inflammation and sepsis” Hospices Civils de Lyon – Université Claude Bernard Lyon I, Lyon, France; 4 Hospices Civils de Lyon – Immunology Laboratory, Hôpital Edouard Herriot, Lyon, France; University of Leuven, Rega Institute, Belgium

## Abstract

**Objectives:**

Septic syndromes are the leading cause of death in intensive care units. They are characterized by the development of immune dysfunctions such as endotoxin tolerance (ET), whose intensity and duration are associated with increased risk of nosocomial infections and mortality. Alarmins S100A8 and S100A9 have been shown to be increased after septic shock. Importantly, a delayed S100A9 mRNA increase predicts hospital-acquired infection in patients. The aim of this study was to investigate the regulation of S100A8 and S100A9 mRNA expression in an *ex vivo* model of ET.

**Subjects and Measurements:**

ET was reproduced *ex vivo* by priming healthy peripheral blood mononuclear cells (number of donors  = 9 to 10) with low-dose endotoxin (2 ng/ml) before stimulation with high dose endotoxin (100 ng/ml). S100A8 and S100A9 mRNA levels were measured by quantitative real-time polymerase chain reactions.

**Main Results:**

ET was established by observing decreased TNFα and increased IL-10 transcriptomic responses to two subsequent endotoxin challenges. Interestingly, ET was associated with increased S100A8 and S100A9 mRNA expression *ex vivo*. We showed that IL-10 played a role in this process, since S100A8 and S100A9 mRNA increases were significantly abrogated by IL-10 blockade in the model. Conversely, treatment with rIFN-γ, a pro-inflammatory and immunostimulating molecule known to block ET induction, was able to restore normal S100A8 and S100A9 mRNA in this model.

**Conclusions:**

In this *ex vivo* model, we observed that S100A8 and S100A9 mRNA expression was significantly increased during ET. This reproduced *ex vivo* the observations we had previously made in septic shock patients. Interestingly, IL-10 blockade and rIFN-γ treatment partially abrogated S100A8/A9 mRNA increases in this model. Pending confirmation in larger, independent clinical studies, these preliminary results suggest that S100A8 and S100A9 mRNA levels might be used as surrogate markers of ET and as stratification tools for personalized immunotherapy in septic shock patients.

## Introduction

Sepsis is a major health care challenge and represents one of the leading causes of death in intensive care units. It affects 750,000 people annually in the United States, and its incidence is increasing, partially because of the aging population. In its most severe form, septic shock, mortality can reach 50%, and limited progress has been made in patient management despite the large number of studies on this topic [1], [2].

The community's understanding of sepsis pathophysiology has been challenged in recent years. In particular, alterations of the immune system are now considered an additional organ dysfunction to be diagnosed and treated in sepsis. Indeed, in septic shock patients who survive the initial inflammatory storm, the immune response often evolves toward a state of immunosuppression, which contributes to increased mortality and severe secondary hospital-acquired infections [2], [3]. One of the current trends in the therapeutic management of sepsis is treatment of this immune dysfunction in an attempt to improve the patient prognosis. However, as there are no clinical signs of sepsis-induced immune dysfunctions, its diagnosis is difficult and is currently based on laboratory techniques that are impractical for clinicians. As a result, there is now a strong need for the development of tools to quickly assess the immune status of patients, to select patients who are most likely to benefit from immunostimulatory treatments, and to monitor the effectiveness of these treatments. Moreover, a better understanding of the pathophysiology of sepsis-induced immune dysfunctions is mandatory for the development of these tests and new treatments.

S100A8 and S100A9 proteins are part of the alarmins family. These proteins are constitutively expressed in neutrophils (they represent 45% of cytosolic proteins) and are induced in other cell types such as monocytes and endothelial cells [4]. Their structure is composed of two calcium binding loops, and they can combine to form dimers, the best known being the heterodimer S100A8/A9, also known as calprotectin. Many immune functions have been described for S100A8 and S100A9 proteins, including pro- and anti-inflammatory properties, activation of neutrophil migration and endothelial cells, antibacterial activity and cell death induction [4]–[11].

S100A8 and S100A9 have been shown to be increased after septic shock, both in terms of the protein and mRNA levels [12], [13]. Of significance, we observed in a previous study that delayed S100A9 mRNA increase predicted hospital-acquired infection in patients with septic shock [13]. We therefore postulated that this increased S100A9 mRNA observed after septic shock may be related to the development of immune alterations in these patients, knowing that no previous study investigated this aspect.

In the current study, we tested this hypothesis by using the model of endotoxin tolerance, which partially reproduces *ex vivo* the monocytic part of sepsis-induced immunosuppression. Indeed, monocytes from septic shock patients are characterized by a reduced TNF production when stimulated *ex vivo* by LPS (lipopolysaccharide), in association with an increased IL-10 production in comparison with cells from healthy controls. This immune alteration is called endotoxin tolerance and can be reproduced *ex vivo.* In this model, the initial exposure to low doses of endotoxin causes cellular reprogramming towards an anti-inflammatory state, in which TNFα production by monocytes decreases in response to a second exposure to LPS, while the production of IL-10 increases [14].

Therefore, the goal of our study was to study the regulation of S100A8 and S100A9 mRNA levels in an *ex vivo* model of endotoxin tolerance, which is known to partially reproduce sepsis-induced innate immune alterations.

## Materials and Methods

### Healthy volunteers

Citrated blood from healthy adult blood donors (80% men, median age 48 yrs) was obtained from the French blood bank, EFS (Etablissement Français du Sang) and was then used immediately. For each experiment, 9 to 10 different donors (one unit per donor), were tested, depending on the studied conditions. In accordance with EFS standardized procedures for blood donation, informed consent was obtained from healthy volunteers, and personal data relative to blood donors were rendered anonymous at the time of blood donation and before blood transfer to our research lab.

### Isolation of PBMCs and cell culture conditions

PBMCs (peripheral blood mononuclear cells) were obtained from fresh whole blood by Ficoll-Paque density gradient centrifugation (GE Healthcare, Uppsala, Sweden) and washed with sterile PBS (phosphate buffered saline) (Invitrogen, Carlsbad, CA, USA). The remaining red blood cells were lysed. Moreover, flow cytometric analysis of cells purified by using this technique confirmed the absence of neutrophils among PBMCs (data not shown).

PBMCs were adjusted to 2×10^6^ cells/ml and cultured in very low attachment 24-well plates in X-Vivo 20 medium (Lonza, Verviers, Belgium) at 37°C and 5% CO2. All the experiments were done in triplicate.

Lipopolysaccharide was purchased from Sigma-Aldrich and was a mix from *Escherichia coli* O111:B4, O55:B5 and O127:B8 (Sigma-Aldrich, Deisenhofen, Germany).

**Figure 1:**
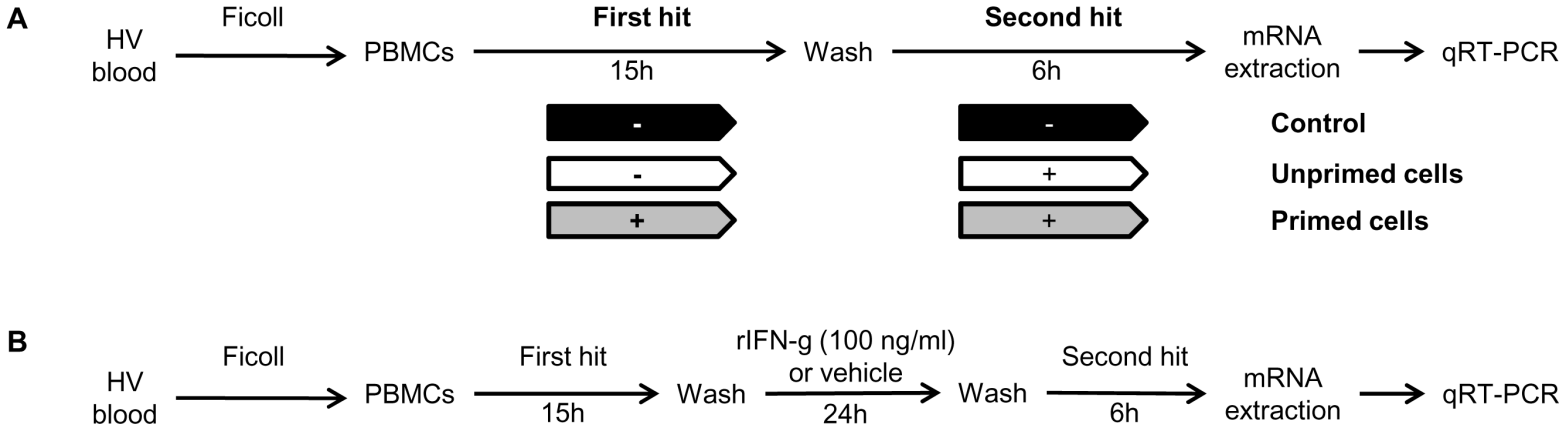
Double hit model with LPS. Figure 1A. Diagram of the model of endotoxin tolerance used in our study. Peripheral blood mononuclear cells (PBMCs) were obtained from fresh whole blood from healthy volunteers (HV) by Ficoll-Paque density gradient centrifugation. Endotoxin tolerance was reproduced *ex vivo* by priming PBMCs with low-dose endotoxin (2 ng/ml) before stimulation with high dose endotoxin (100 ng/ml). S100A8 and S100A9 mRNA levels were measured by quantitative real-time polymerase chain reactions. Figure 1B. Diagram of immune-stimulating therapy model during endotoxin tolerance.

In this *ex vivo* model of endotoxin tolerance, PBMCs were first cultured for 15 hours without (control group and unprimed cells) or with 2 ng/ml LPS (primed cells). After two washing steps, the PBMCs were incubated a second time for 6 hours without (control group) or with 100 ng/ml LPS (unprimed and primed cells – Fig. 1A). In this model, when specified in the text, anti-IL-10 neutralizing antibody (Human IL-10 MAb Clone 25209, 100 ng/ml; R&D systems, Minneapolis, USA) was added during both incubation steps. Finally, when studying the effects of recombinant human IFN-γ, an additional incubation phase with rIFN-γ1b (100 ng/ml; Boehringer, Ingelheim, Austria) or vehicle was performed between the two incubations with LPS (Fig. 1B).

At the end of the experiments, the cells were centrifuged. The resulting cell pellet was lysed and then frozen at −20°C before performing messenger RNA (mRNA) extraction and quantitative real-time polymerase chain reactions (qRT-PCR).

### mRNA extraction and qRT-PCR

Total RNA was extracted from PBMCs using RNeasy Mini kit (Qiagen, Hilden, Germany) according to the manufacturer's instructions. Residual genomic DNA was digested using the RNase-Free DNase set (Qiagen, Hilden, Germany). Each sample was quantified using Qubit Fluorometric Quantitation (Invitrogen, Carlsbad, CA, USA), and 200 ng of total RNA was reverse transcribed into cDNA using the SuperScript VILO system (Invitrogen, Carlsbad, CA, USA) according to the manufacturer's instructions. RNA quality was assessed on a Bioanalyser (Agilent) according to the manufacturer's instructions. Tested samples all had a RNA integrity number (RIN) greater than 7.3, illustrative of a good RNA quality.

Models of endotoxin tolerance are characterized by a reduced expression of genes known as “tolerizable” in association with an increased expression of genes known as “non-tolerizable” following a second stimulation with LPS [15]–[17]. We chose to validate our model by studying the expression of the single most illustrative gene for each group: TNFα (tolerizable) and IL-10 (non-tolerizable).

The mRNA expression of TNFα, IL-10, S100A8, S100A9, Suppressor of cytokine signaling 3 (SOCS3) and the reference gene peptidylpropylisomerase B (PPIB), which encodes for cyclophilin B, were investigated using quantitative real-time PCR [18] (Table 1). Briefly, PCR reactions were performed using a LightCycler480 instrument with the associated SYBR Green I Master Mix, according to the recommendations of the supplier (Roche, Mannheim, Germany), as well as specific cDNA standards and primer mixes. Thermocycling was performed in a final volume of 20 µl containing 0.5 µM of each required primer. PCR was performed with an initial 5 minute denaturation step at 95°C, followed by 40 cycles of a touchdown PCR protocol (15 secs annealing at 68–58°C, and 15 secs extension at 72°C). The LightCycler480 was used to determine the crossing point for each sample. Relative standard curves describing the PCR efficiency of the target or PPIB genes were created and used to perform efficiency-corrected quantification with the LightCycler software version 1.5.0 SP4. The results were expressed as a number-of-copy-concentration ratio between the target gene mRNA and PPIB mRNA, as it has been done in previous studies with human peripheral blood [13], [19].

**Table 1:** Quantitative PCR and primers.

Gene symbol	GenBank	Primer sequences a
PPIB	NM_000942	5′-ACGCAACATGAAGGTGCTC-3′
		5′-CATCTCCAATTCGTAGGTCAAA-3′
TNF	NM_000594	5′-CCTTGGTCTGGTAGGAGACG-3′
		5′-TCTCTAATCAGCCCTCTGGC-3′
IL-10	NM_000572	5′-AAGGGGCTGGGTCAGCTAT-3′
		5′-AGAACCAAGACCCAGACATCAA-3′
S100A8	NM_002964	5′-ATTTCCATGCCGTCTACAGG-3′
		5′-CACCAGAATGAGGAACTCCT-3′
S100A9	NM_002965	5′-TCAAAGAGCTGGTGCGAAAA-3′
		5′-AACTCCTCGAAGCTCAGCTG-3′
SOCS3	NM_003955	Primers purchased from Search-LC (Heidelberg, Germany)

a Top sequence is forward primer, bottom is reverse.

### Statistics

Results are presented as fold change relative to the control group, and as medians and interquartile range (IQR). The non-parametric Wilcoxon paired test was used to assess variations between cell culture conditions. Correlation was assessed using Spearman correlation test. Statistical analyses were performed using Prism 5.02 for Windows (GraphPad Software, La Jolla, CA, USA). A *p* value <0.05 was considered statistically significant.

## Results

### Endotoxin tolerance is associated with increased S100A8 and S100A9 mRNA expression

We first investigated the regulation of S100A8 and S100A9 mRNA expression in an *ex vivo* model of endotoxin tolerance (Figure 1A).

**Figure 2:**
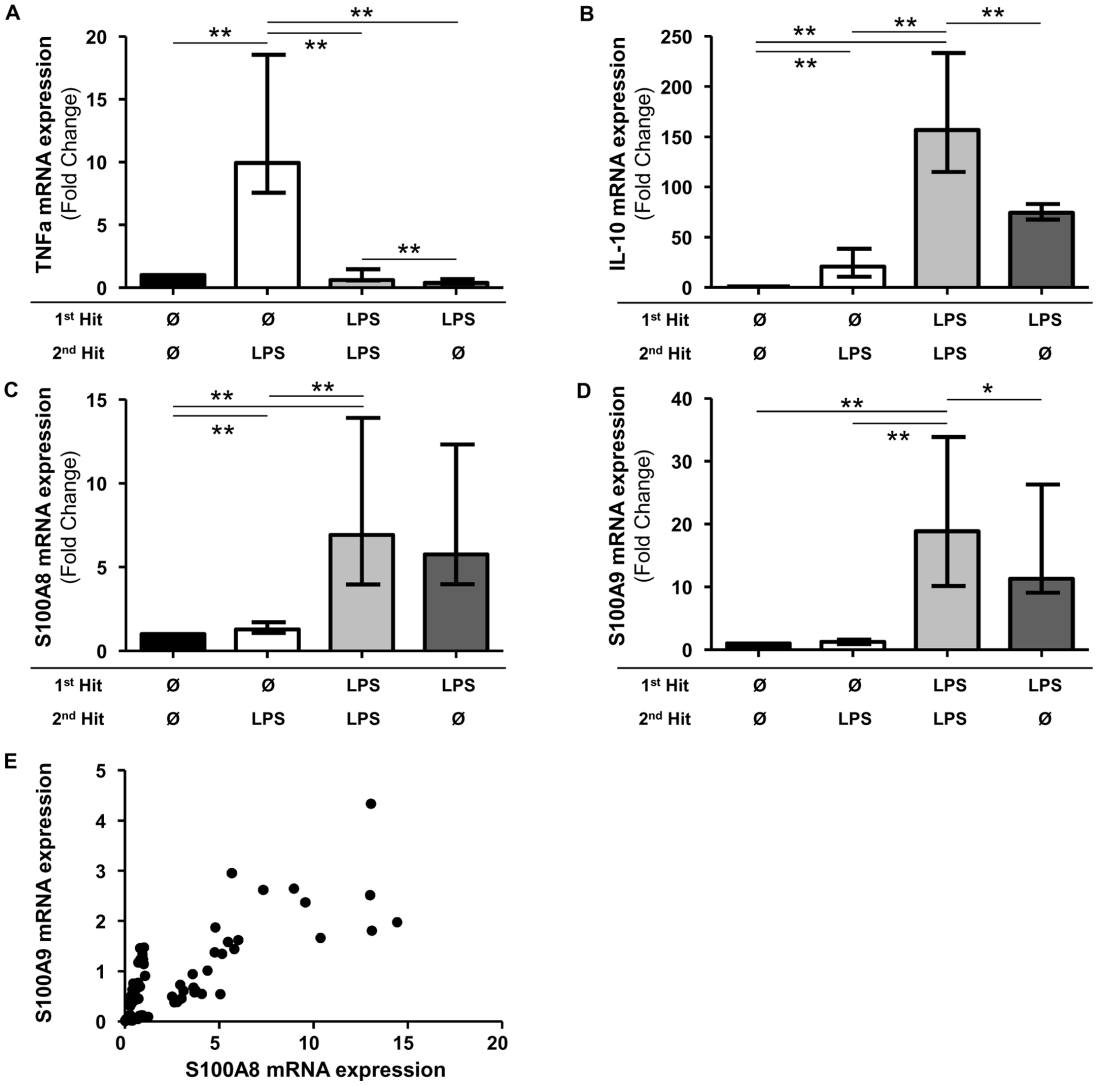
Endotoxin tolerance is associated with decreased TNFα and increased IL-10, S100A8 and S100A9 mRNA expressions. Figures 2A, B, C and D. Messenger RNA (mRNA) level of tolerizable gene (TNFα) and non-tolerizable genes (IL-10, S100A8 and S100A9) in an *ex vivo* model of endotoxin tolerance. The mRNA level was normalized to that of the reference gene peptidylpropylisomerase B (PPIB) and then compared to the control group. Black columns represent controls (cells without any lipopolysaccharide (LPS)), white columns represent LPS-unprimed cells (only stimulated once with 100 ng/ml LPS), light grey columns represent LPS-primed cells (stimulated twice: 2 ng/ml followed by 100 ng/ml) and dark grey columns represent cells stimulated once with 2 ng/ml LPS without any second stimulation. **<0.01, *<0.05, Wilcoxon signed-rank test. Median (+/− interquartile range) data from 10 independent experiments are given. Figure 2E. Correlation between S100A8 mRNA expression (x-axis) and S100A9 mRNA expression (y-axis) in the endotoxin tolerance model. S100A8 and S100A9 mRNA levels were normalized to that of PPIB. Data were obtained from the 10 independent experiments described above.

As expected, in response to a second LPS challenge, the mRNA level of TNFα in our model was increased in unprimed cells (median [IQR] of fold-change vs. non-stimulated cells: 9.9 [7.6–18.5]), while it was collapsed in LPS-primed cells (0.6 [0.6–1.5], *p* = 0.0059) (Fig. 2A). Conversely, the mRNA levels of IL-10 were higher in primed cells compared to unprimed cells (156.8 [115.1–233.4] vs. 20.8 [10.7–38.5], respectively, *p* = 0.0020). Thus, the inability to induce TNFα in response to a second LPS challenge combined with an increased induction of IL-10 confirmed the development of an endotoxin tolerance state in our model.

Interestingly, while a small but significant increase was observed after a single LPS stimulation (control vs. unprimed, *p*<0.001), the S100A8 mRNA levels were largely and significantly increased in LPS-primed cells compared to unprimed cells (6.9 [4.0–13.9] vs. 1.3 [1.1–1.7], respectively, *p* = 0.0020, Fig. 2C). As it was observed for S100A8, the S100A9 mRNA levels followed the same regulation pattern as they were increased in LPS-primed cells compared to unprimed cells (18.9 [10.2–33.9] vs. 1.3 [0.9–1.6], respectively, *p* = 0.0020, Fig. 2D). Finally, in this model, we observed that mRNA expression of S100A8 and S100A9 were highly correlated (r = 0.8464, *p*<0.0001, Fig. 2E).

Thus, S100A8 and S100A9 mRNA expression is increased in this model of endotoxin tolerance in parallel with IL-10 mRNA.

### IL-10 plays a partial role in S100A8 and S100A9 increase during ex vivo endotoxin tolerance

As previously published articles showed that IL-10 plays a role in increased S100A8 and S100A9 expression [20], [21] and as we observed a parallel regulation of S100A8 and S100A9 and IL-10 mRNA expression in our model, we next explored whether IL-10 could modulate the expressions of S100A8 and S100A9 in our *ex vivo* model of endotoxin tolerance.

**Figure 3:**
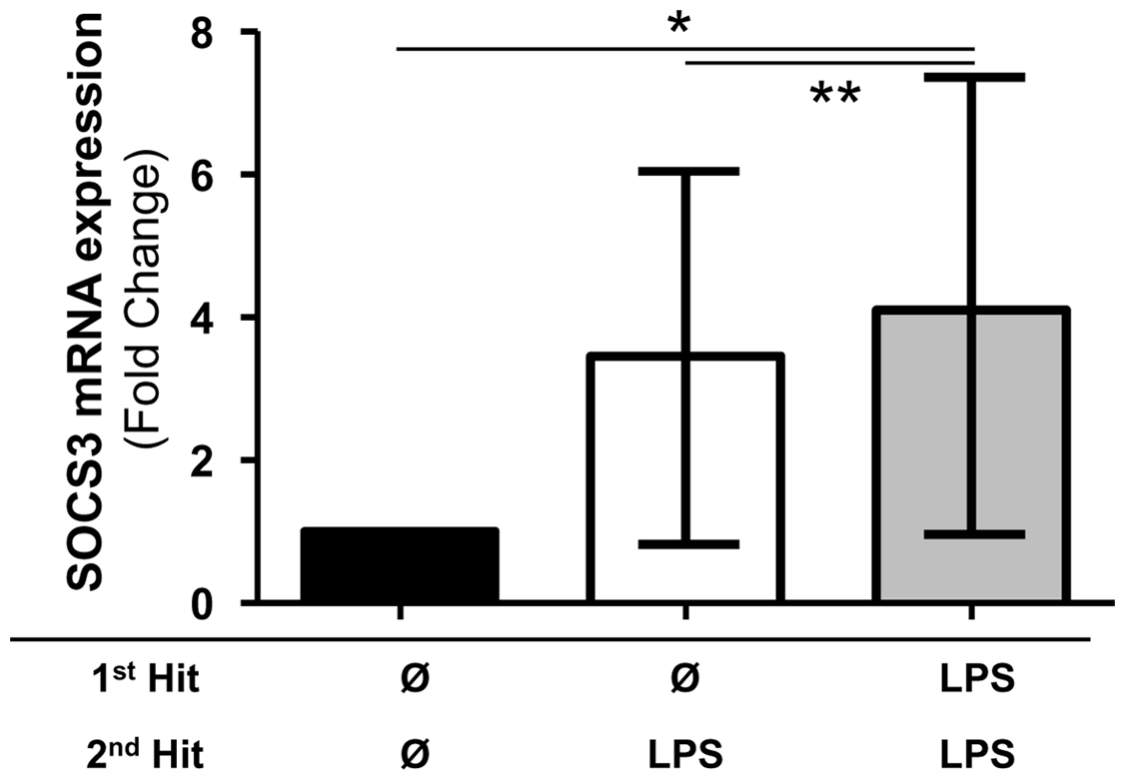
Endotoxin tolerance is associated with increased SOCS3 mRNA expression. Messenger RNA (mRNA) level of SOCS3 in an *ex vivo* model of endotoxin tolerance. The mRNA level was normalized to that of the reference gene peptidylpropylisomerase B (PPIB) and then compared to the control group. Black columns represent controls (cells without any lipopolysaccharide (LPS)), white columns represent LPS-unprimed cells (only stimulated once with 100 ng/ml LPS) and grey columns represent LPS-primed cells (stimulated twice: 2 ng/ml followed by 100 ng/ml). **<0.01, *<0.05, Wilcoxon signed rank test. Median (+/− interquartile range) data from 9 independent experiments are given.

First, we evaluated the regulation of SOCS3 mRNA expression in this model, knowing that SOCS3 is induced when IL-10 intracellular pathway is activated. We observed that SOCS3 mRNA was significantly increased in cells after a double LPS stimulation in comparison with non-stimulated cells (4.1 [1.0–7.4] vs. 1.0 [1.0–1.0], respectively, *p* = 0.0391) or cells stimulated once with LPS (4.1 [1.0–7.4] vs. 3.5 [0.8–6.0], respectively, *p* = 0.0078, Fig. 3).

**Figure 4:**
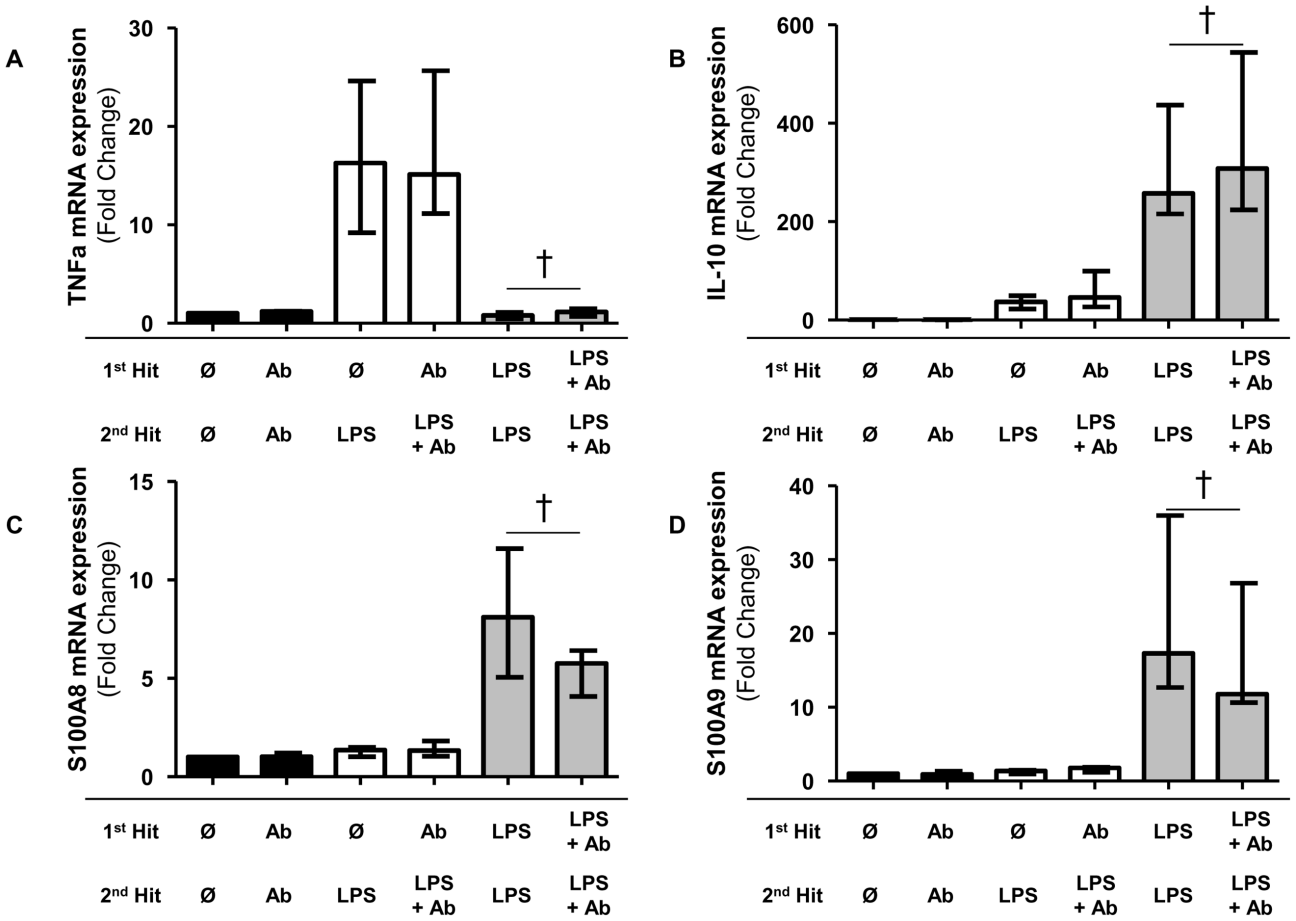
S100A8 and S100A9 mRNA expression increases were partially abrogated by IL-10 blockade. Messenger RNA (mRNA) level of TNFα, IL-10, S100A8 and S100A9 in an *ex vivo* model of endotoxin tolerance in the presence or absence of anti-IL-10 antibodies (Ab). Anti IL-10 Ab was used at 100 ng/ml. The mRNA level was normalized to that of the reference gene peptidylpropylisomerase B (PPIB) and then compared to the control group. Black columns represent controls (cells without any lipopolysaccharide (LPS)), white columns represent LPS-unprimed cells (only stimulated once with 100 ng/ml LPS) and grey columns represent LPS-primed cells (stimulated twice: 2 ng/ml followed by 100 ng/ml). †p<0.01, Wilcoxon paired test. Median (+/− interquartile range) data from 9 independent experiments are given.

We then blocked IL-10 during both LPS stimulations with anti-IL-10 neutralizing antibodies. Of note, after incubation with anti-IL-10 antibodies, we observed a slight increase in TNF and IL-10 mRNA levels in response to a second LPS stimulation (*p*<0.01, Fig. 4A and 4B).

An interesting observation was that the induction of S100A8 mRNA levels in response to a second LPS stimulation (primed cells) was significantly decreased in the presence of IL-10 blocking antibodies (8.1 [5.1–11.6] vs. 5.8 [4.1–6.4], respectively, in primed cells incubated without or with anti-IL-10 antibodies, *p*<0.01) (Fig. 4C).

Similarly, the induction of S100A9 mRNA levels in response to a second LPS stimulation (primed cells) was significantly decreased in the presence of anti-IL-10 antibody (17.3 [12.7–36.0] vs. 11.8 [10.6–26.8], respectively, in primed cells incubated without or with IL-10 antibody, *p*<0.01) (Fig. 4D).

Thus, our results show that induction of S100A8 and S100A9 mRNA expression after 2 LPS stimulations was significantly decreased in the presence of anti-IL-10 neutralizing antibody suggesting that IL-10 may play a part in the induction of S100A8 and S100A9 mRNA in this model of endotoxin tolerance.

### S100A8 and S100A9 increase is partially abolished by rIFN-γ

As our first results showed that S100A8/A9 mRNA levels were induced during endotoxin tolerance *ex vivo*, we wondered whether the reversal of endotoxin tolerance by an immunostimulatory cytokine could be associated with decreased S100A8 and S100A9 mRNA expression.

Indeed, recombinant human IFN-γ has shown its ability to restore monocytic functions both in vitro and in patients [22]. We thus tested the effects of IFN-γ in our model by adding an incubation phase with this immunostimulating molecule between the two LPS incubations steps (Fig. 1B).

**Figure 5:**
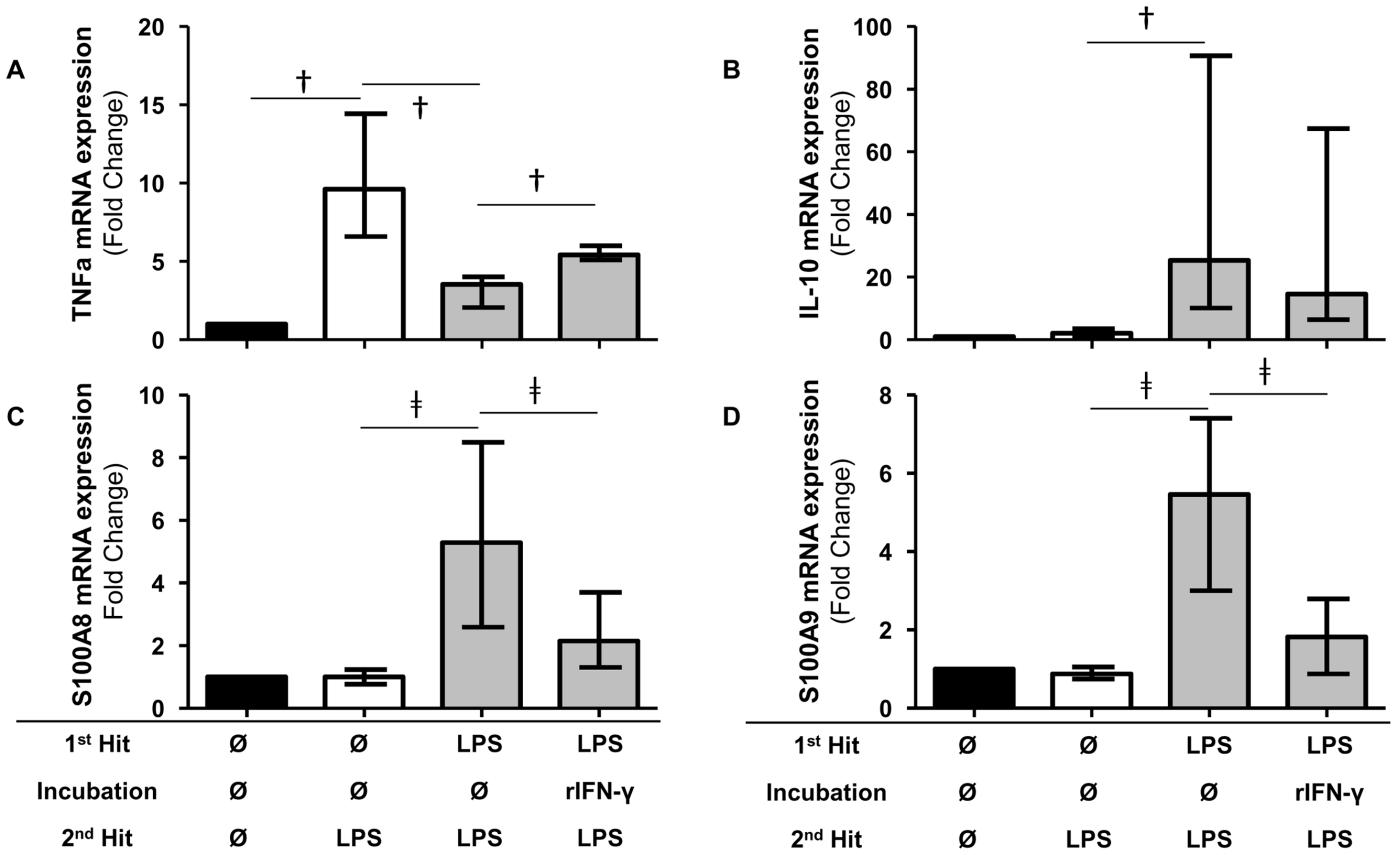
S100A8 and S100A9 mRNA level increases were significantly abrogated by rIFN-γ in endotoxin tolerance model. Messenger RNA (mRNA) level of TNFα, IL-10, S100A8 and S100A9 in an *ex vivo* model of endotoxin tolerance. The mRNA level was normalized to that of the reference gene peptidylpropylisomerase B and then compared to the control group. Black columns represent controls (cells without any lipopolysaccharide (LPS)), white columns represent LPS-unprimed cells (only stimulated once with 100 ng/ml LPS) and grey columns represent LPS-primed cells (stimulated three times: 2 ng/ml LPS followed by vehicle or IFN-γ followed by 100 ng/ml LPS). †p<0.01, ‡p<0.05, Wilcoxon paired test. Median (+/− interquartile range) data from 10 independent experiments are given.

Interestingly and as expected, IL-10 mRNA expression decreased in the rIFN-γ-treated vs. vehicle-treated primed cells (14.5 [6.4–67.4] vs. 25.3 [10.1–90.7], respectively, Fig. 5B) while TNFα mRNA expression increased in rIFN-γ vs vehicle treated-primed cells (5.4 [5.1–6.0] vs. 3.5 [2.1–4.0], respectively, *p* = 0.001, Fig. 5A). This shows that ET is partially reversed by rIFN-γ.

Most importantly, we observed that rIFN-γ treatment in LPS-primed cells was associated with a significantly decreased mRNA expression of S100A8 compared with untreated primed cells (2.2 [1.3–3.7] vs. 5.3 [2.6–8.5], respectively, *p* = 0.002) (Fig. 5C).

Similarly, the expression of S100A9 mRNA was significantly decreased in rIFN-γ-treated vs. vehicle-treated primed cells (1.8 [0.9–2.8] vs. 5.4 [3.0–7.4], respectively, *p* = 0.002) (Fig. 5D).

In conclusion, we observed that reversal of endotoxin tolerance *ex vivo* by rIFN-γ treatment was associated with a restoration of normal alarmin responses in our model. This suggests that S100A8/A9 mRNA levels may be considered surrogate markers of ET *ex vivo*.

## Discussion

Sepsis-induced immunosuppression, characterized by the development of endotoxin tolerance, is believed to be responsible for the increased risk of nosocomial infections and mortality [3], [23], [24]. Therapeutic management of this immunosuppression may improve the prognosis of patients with septic shock [25]. Better understanding and a description of the pathophysiological mechanisms of sepsis-induced immunosuppression are important for such therapies and the development of associated biomarkers for patient stratification based on their immune status.

Beside the traditional detection of pathogens by pathogen-associated molecular patterns such as LPS, damaged tissues release a group of endogenous molecules named alarmins or danger-associated molecular patterns (DAMPs), such as high mobility group box 1 (HMGB1), histones, DNA, heat shock proteins and some S100 proteins. These endogenous molecules are also detected by receptors that induce inflammatory responses.

The alarmins S100A8 and S100A9 are increased in many acute or chronic inflammatory diseases in patients. The first increase was described in 1980 [26]. In rheumatoid arthritis, high concentrations of these proteins have been found in inflamed tissue, whereas their levels are low in healthy individuals [27]. As a result, elevations of these proteins have been described in many diseases such as inflammatory bowel disease, transplant rejection, acute pancreatitis, peritonitis, appendicitis and myocardial infarction [28]–[33].

The role of these alarmins in the pathophysiology of inflammation in general and of sepsis in particular remains unclear and controversial [31], [34]. Several studies have recently described their increase during sepsis. Patients with severe sepsis have an increased serum S100A8/A9 concentration [31]. Our research group has also shown that S100A9 mRNA is increased after septic shock, and that a delayed increase in the expression of S100A9 was associated with hospital-acquired infections after septic shock [13]. Moreover, the preliminary results on a small cohort of 17 patients with septic shock showed that plasma levels of S100A8 may be increased in patients who do not survive, and that S100A8 decreases during the recovery phase [12]. Literature results support the fact that S100A8/A9 proteins and/or mRNA levels are increased in septic patients, and there may be a link between a high elevation and an increased risk of death or nosocomial infections.

Therefore, S100A8/A9 proteins and/or mRNA expression may be regulated in accordance with the development of immune dysfunctions in sepsis. This hypothesis has never been tested in the literature so far. In the current study, we thus evaluated the regulation of S100A8 and S100A9 mRNA expressions in an *ex vivo* model of sepsis-induced immune dysfunctions (endotoxin tolerance). This model was set up in previous studies from our group. In these experiments, we identified transcripts which expressions were altered during endotoxin tolerance and could be restored by rIFN-γ [16], [17]. However, none of this previous work specifically focused on the regulation of S100A8/A9 mRNA expressions in this model.

Interestingly, we showed that S100A8 and S100A9 mRNA levels were increased in an *ex vivo* model of endotoxin tolerance. These *ex vivo* results could be related with those previously obtained in septic shock patients, who were known to develop such innate immune dysfunctions and who presented with increased concentrations of these alarmins.

These results are the first to show such large increases in S100A8 and S100A9 mRNA in a model of 2 subsequent LPS stimulations. Previous studies in models of a single endotoxin challenge showed that a single LPS stimulation induced S100A8/A9 production in human monocytes [35] or bone marrow cells [36]. This is consistent with our results. However, in our study, the increase of S100A8/A9 after a single LPS challenge was minimal compared with their elevation after 2 LPS stimulations.

In our model, S100A8 and S100A9 mRNA increases were associated with an increased expression of IL-10. Plasma IL-10 is known to be increased in septic shock [1] and contributes to the dysfunction of innate immunity [37].

Moreover, the literature suggests that IL-10 is involved in the regulation of S100A8 and S100A9 in response to LPS. For example, S100A8 and S100A9 are induced in human monocytes by double strand RNA, and this induction depends on IL-10 [21]. It has been also recently shown that IL-10 promotes secretion of S100A8/A9 from monocytes [38]. Furthermore, it has been shown that IL-10 synergizes with LPS to increase mRNA and secreted S100A8 levels of murine macrophages [20]. However, as the structure and function of S100A8 and S100A9 can vary according to species (the hinge region of murine S100A8 contributes to chemotactic activity, in contrast to that of human S100A8 [39] and murine S100A8 seems to functionally correspond to the human S100A12 protein which does not exist in mice [40], [41]), the observations made on murine models have to be confirmed on human models.

In this study, we have likewise shown that SOCS3 mRNA level, which is known to be induced by IL-10, followed the same pattern of expression as IL-10 and S100A8/A9 in our model. Moreover, we observed that increased S100A8 and S100A9 mRNA after two LPS stimulations were reduced when IL-10 was blocked. Although an appropriate control for IL-10 blockage would be required, these results suggest that IL-10 intracellular pathway is activated during endotoxin tolerance in our model and may play a role in S100A8/A9 induction in this model.

This partial participation of IL-10 in S100A8/A9 regulation in our model could be explained by the existence of other regulators, such as TNFα, for S100A8 [42]. Similarly, other S100A8 and S100A9 regulators, such as IL-6 and TNFα, have been identified on human monocytic leukemia cells [43]. Finally, glucocorticoids increased constitutive levels of S100A8 and S100A9 mRNA in human monocytes [44]. The role of these molecules in the regulation of S100A8/A9 mRNA levels in our model requires further investigation.

As S100A8 and S100A9 are upregulated during endotoxin tolerance, we then wanted to verify that they were down-regulated when the endotoxin tolerance was reversed. This was done using IFN-γ, which plays a major role in activation of monocytes [45]. Moreover, the use of IFN-γ in septic patients with low monocytic HLA-DR expression increased the expression of HLA-DR and restored TNFα secretion induced by LPS [22]. In the latter study, the restoration of monocyte function resulted in the elimination of sepsis in most patients.

Importantly, our results show that the decrease of endotoxin tolerance is associated with decreased mRNA S100A8 and S100A9 expression after treatment with IFN-γ *ex vivo*. This result further confirms that S100A8 and S100A9 mRNA levels are regulated in parallel with endotoxin tolerance (increased during ET induction, decreased after ET reversal).

Another important aspect of our work was the demonstration that the mRNA expressions of S100A8 and S100A9 were closely correlated *ex vivo*. These results are consistent with those obtained by other research groups [21], [46]. This suggests that the single measurement of either S100A8 or S100A9 mRNA levels may be useful for evaluating their response and for use as a potential biomarker in sepsis. With that said, this needs to be confirmed in a clinical study evaluating both S100A8 and S100A9 mRNA levels in patients in regard with clinical outcomes.

This is important because, as there are no clinical signs of immune dysfunctions or laboratory tools routinely available to diagnose immunosuppression, it is essential to develop novel biomarkers of immunosuppression after sepsis.

Sepsis-induced immunosuppression can be reversed with immunostimulatory treatments, such as IFN-γ or GM-CSF [22], [47], [48]. Monitoring the immune status of patients with sepsis could allow selection of the population most likely to benefit from such treatment [1], [49]. Notably, Meisel *et al.* have used the expression level of mHLA-DR to guide the prescription of GM-CSF in patients with severe sepsis or septic shock [47]. Decreased HLA-DR expression on monocytes is a reference marker for immunosuppression in critically ill patients [50]. The routine use of this test however is limited by its lack of availability, duration and the difficulty of achieving protocol standardization. One alternative could be molecular biology tests, whose standardization is facilitated by the development of fully automated platforms.

On the whole in this experimental study, we showed that S100A8/A9 mRNA levels vary in parallel with the induction of endotoxin tolerance *ex vivo*. This suggests that the mRNA expression level of these alarmins could be surrogate markers of sepsis-induced immune dysfunctions. This could be related with clinical observations including our own that show a link between increased S100A8/A9 levels after sepsis and an increased risk of death or nosocomial infections [12], [13].

There are some limitations to our study. First, it would have been interesting to assess the pathophysiological involvement of these alarmins in endotoxin tolerance. Vogl *et al.* showed that S100A8/S100A9 was a ligand for Toll-like receptor 4 (TLR4) and seems to amplify the inflammatory response of bone marrow cells in response to LPS [36]. The binding of this ligand to TLR4 leads to activation of intracellular signaling pathways through MyD88, ERK, p38 MAPK, and PKC. S100A9 induces a stronger activation of NF-KB but a lower cytokine secretion than LPS [51]. Overall, the pathophysiological role of these alarmins varies considerably from one model to another, and clarification of this will require dedicated studies.

Secondly, we focused on mRNA expression, as we did in our initial clinical study [13]. To note, we measured S100A8/A9 protein release in the supernatants obtained after cell culture in our model (Data not shown). However, we did not observe any difference between the conditions tested. This study could thus be completed by the evaluation of intracellular concentrations of S100A8/A9 in our model.

In addition, the partial role of IL-10 in the regulation of S100A8/A9 mRNA levels in this model needs to be confirmed by performing specific experiments targeting IL-10 intracellular pathway or in mouse models, as well as by using appropriate controls of IL-10 blockage in the model of endotoxin tolerance.

Since we did not perform any experiment on a specifically purified cell type, we cannot ascertain in which cell type is S100A8/A9 induced. Data from the literature suggest that S100A8/A9 is not inducible in lymphocytes [52] and monocytes are known to be the main target of LPS in the model of endotoxin tolerance. Although this deserves further experiments, these data suggest that the observed changes in S100A8/A9 expression may mainly occur in monocytes.

Finally, the regulation of S100A8/A9 mRNA expression with regard to the development of endotoxin tolerance now requires clinical validation.

## Conclusions

To conclude, we observed that S100A8 and S100A9 mRNA expression increased during endotoxin tolerance. This corresponds to the clinical data, as they are increased in patients with septic shock and those who will develop hospital-acquired infections. We also showed that IL-10 is involved in the regulation of their expression. Moreover, rIFN-γ treatment partially abrogated the increase of S100A8/A9 mRNA. Our results suggest that S100A8 and S100A9 mRNA levels may be used as surrogate markers of endotoxin tolerance after sepsis. After confirmation in a larger study clinical study, they may help to identify septic patients at risk of deleterious outcomes (death/nosocomial infections) and may be used as a stratification tool for personalized immunotherapy in patients with septic shock.
